# Analyses of Long Non-Coding RNA and mRNA profiling using RNA sequencing during the pre-implantation phases in pig endometrium

**DOI:** 10.1038/srep20238

**Published:** 2016-01-29

**Authors:** Yueying Wang, Songyi Xue, Xiaoran Liu, Huan Liu, Tao Hu, Xiaotian Qiu, Jinlong Zhang, Minggang Lei

**Affiliations:** 1 Key Lab of Swine Genetics and Breeding of Ministry of Agriculture, Key Lab of Agricultural Animal Genetics, Breeding and Reproduction of Ministry of Education, College of Animal Science and Technology, Huazhong (Central China) Agricultural University, Wuhan, Hubei, PR China; 2 National Animal Husbandry Services Ministry of Agriculture, Beijing, PR China

## Abstract

Establishment of implantation in pig is accompanied by a coordinated interaction between the maternal uterine endometrium and conceptus development. We investigated the expression profiles of endometrial tissue on Days 9, 12 and 15 of pregnancy and on Day 12 of non-pregnancy in Yorkshire, and performed a comprehensive analysis of long non-coding RNAs (lncRNAs) in endometrial tissue samples by using RNA sequencing. As a result, 2805 novel lncRNAs, 2,376 (301 lncRNA and 2075 mRNA) differentially expressed genes (DEGs) and 2149 novel transcripts were obtained by pairwise comparison. In agreement with previous reports, lncRNAs shared similar characteristics, such as shorter in length, lower in exon number, lower at expression level and less conserved than protein coding transcripts. Bioinformatics analysis showed that DEGs were involved in protein binding, cellular process, immune system process and enriched in focal adhesion, Jak-STAT, FoxO and MAPK signaling pathway. We also found that lncRNAs *TCONS_01729386* and *TCONS_01325501* may play a vital role in embryo pre-implantation. Furthermore, the expression of *FGF7*, *NMB, COL5A3, S100A8* and *PPP1R3D* genes were significantly up-regulated at the time of maternal recognition of pregnancy (Day 12 of pregnancy). Our results first identified the characterization and expression profile of lncRNAs in pig endometrium during pre-implantation phases.

Successful implantation of pregnancy is dependent on a complex molecular cross-talk between the conceptus development and the maternal uterus^1^,^2^. The embryo-maternal communication occurs approximately on Day 12 of pregnancy in pigs^3^, and the establishment of implantation in pig is accompanied by a dynamic production of estrogens, progesterone, prostaglandins, adhesion molecules and immunological factors. Porcine conceptuses secrete a large amount of estrogens, primarily 17β-estradiol (E_2_) on Days 11 and 12 and between Days 14 and 18 of pregnancy^4^,^5^,^6^. Estrogens enhance endometrial prostaglandin E_2_ (PGE_2_) production around Day 12 of pregnancy, resulting in sequestering of prostaglandin (PGF_2α_) within the uterine lumen and increasing the PGE_2_: PGF_2α_, which could promote the maintenance of corpus luteum (CL) and progesterone (P_4_) production^7^,^8^. P_4_ could stimulate the secretion of endometrial production required for conceptus development and implantation. Higher PGE_2_ secretion in uterine lumen coincides with the elevated expression of HOXA10 transcription factor which is critical for implantation. A stable adhesion between conceptus and endometrium requires reduction in mucin-1 on the apical surface of epithelium Furthermore, growth factors, cytokines and its receptors are all involved in embryo-maternal interactions^9^,^10^. In response to these factors, the uterine endometrial undergoes dramatic morphological and functional changes to be more easily receptive to the conceptus development^11^. Thus, to increase the rate of successful implantation, it is essential to understand the mechanism of the genetic regulation of endometrial changes.

Some of the identified genes are revealed to have expression changes of different levels in the uterine endometrial during early pregnancy at the time of estrogen release. For example, fibroblast growth factor 7 (*FGF7*)^12^,^13^ and secreted phosphoprotein 1 (*SPP1*)^14^,^15^ are abundantly expressed in uterine endometrial epithelial cells during early pregnancy. Interleukin-1 (IL-1) is identified as a key regulator of inflammatory response that modulates the communication between the maternal endometrium and embryo^16^,^17^. Legumain (*LGMN*) and its inhibitor, namely cystatins 6 (*CST6*) at the maternal-fetal interface, may play an important role in the establishment and maintenance of pregnancy in pigs^18^. In addition to the investigation of single-candidate genes and pathways, many studies have revealed a number of many different patterns of gene expression in uterine endometrial during the process of maternal recognition of implantation and pregnancy by using various technologies. Take for instance, microarrays have been used to describe endometrial gene expression on Days 12^11^,^19^,^20^, 14^21^, 15^11^,^22^ and 16^19^ of the estrous cycle and pregnancy, and on Days 13, 18 and 24 of the attachment and non-attachment sites^23^. Similarly, digital gene expression profiling has been used to described differences in gene expression of endometrial samples collected from Erhualian sows and Landrace × Large White pigs on Day 12 of pregnancy^24^. In addition, systematic studies have been performed by RNA sequencing (RNA-seq) to describe the transcriptome changes in prepuberal gilts (crossbreed of German Landrace and Pitetrain) endometrium on Days 12 and 14 of the estrous cycle and pregnancy^25^,^26^. A further advantage of this whole transcriptome sequencing technology is able to describe unannotated transcriptional activity by identifying numerous noncoding transcripts^27^. Long non-coding RNAs (lncRNAs) have received much attention in the past several years, and are found to play important functional roles in epigenetic regulation, chromatin modification, genomic imprinting, transcriptional control as well as pre- and post- translational mRNA processing^28^. RNA-seq technology has largely leveled up the discovery and analysis of non-coding RNA, and differential methods have been developed to identify novel lncRNAs using RNA-seq data^29^,^30^. Despite the fact that many studies highlight the important roles of lncRNAs in different tissues^30^,^31^,^32^, little is known about the biological function and significance of lncRNAs in uterine endometrial during embryo implantation in pigs.

In this study, we investigated the expression profiles of endometrial tissue on Days 9 (YK9), 12 (YK12) and 15 (YK15) of pregnancy and on Days 12 (YK12K) of non-pregnancy in Yorkshire (YK), and conducted a comprehensive analysis of lncRNAs of endometrial tissue samples by using RNA-seq. The goals of the study were to determine how many and which transcripts (mRNA and lncRNA) are differentially expressed, the relationship between the selected lncRNA with its neighbor mRNA, and to determine which biological processes and pathways are significantly changed by comparing tanscriptomic profiles of endometrial in different pregnant periods. To its credit transcripts information from pig endometrial transcriptomes can be used for further gene expression studies in endometrial tissue, which may help a better understanding of molecular and cellular events that occur in the endometrial during implantation period.

## Results

### RNA Sequencing and identification of mRNA and lncRNAs in Porcine Endometrial

**Figure 1:**
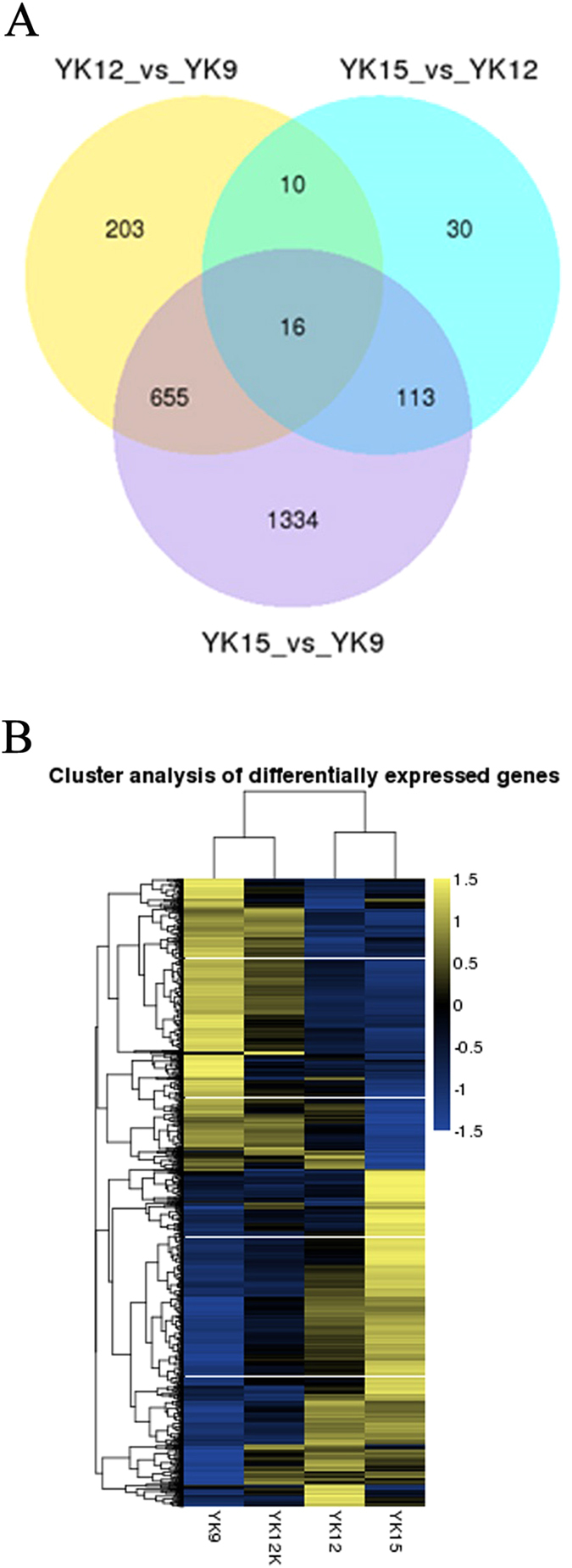
Gene expression profiling and number of differentially expressed genes for endometrium during pre-implantation phase. (**A**) Venn diagram of common differential expression genes in three comparison groups (YK12 vs YK9, YK15 vs YK12 and YK15 vs YK9). (**B**) A hierarchical heat map showing the transformaed expression values for transcript (mRNA and lncRNA). Yellow shows higher expression and blue shows lower espression.

We analyzed the RNA-Seq data from 12 porcine endometrial samples in which 85 to 105 million raw reads and 82 to 99 million clear reads per sample were obtained. 4953 novel lncRNAs were assembled by Cufflinks^33^ and Scripture^27^ (Supplementary Fig. S1). As the identification of transcripts involved immature mRNA fragments, we used four tools, namely Coding Potential Calculator (CPC), Pfam-scan (PFAM), phylogenetic codon substitution frequency (phyloCSF), Coding-Non-Coding-Index (CNCI) to remove potential coding transcripts. Finally, 2805 putative non-coding transcripts were retained (Supplementary Fig. S2). Given a false discovery rate (FDR) of 4% and *q*-value (*P*-adjusted) <0.05, 2,376 (301 lncRNA and 2075 mRNA) differentially expressed genes (DGEs) were obtained from pairwise comparison of samples collected from Yorkshire pigs on Days 9, 12 and 15 of the pregnancy (i.e., YK9 vs YK12; YK12 vs YK15; YK9 vs YK15) and pairwise Day 12 of pregnancy with Day 12 of non-pregnancy (YK12 vs YK12K) (Table 1 and Supplementary Table S1). As shown in Fig. 1A, 16 DEGs were common among three comparisons (1 lncRNA and 15 mRNA). All of the obtained lncRNAs were not previously identified. In addition, a set of 2149 mRNA transcripts were found not to be annotated, over 39% of novel transcripts could be mapped with human NCBI Refseq database, nearly 14% of them mapped in mouse genome and 29% of them were classified as unknown transcripts (Supplementary Table S2). To validate the RNA-seq results, ten genes (*TCONS_01729386, TCONS_01325501, FGF7, NMB, FGF9, VEGFC, VEGFA, Muc1, ESR1 and RBP4*) were chosen for quantitative PCR (qPCR) (Supplementary Table S3). The selected lncRNAs were significantly different expressed at least in one comparison group and its predicted target genes have been reported involved in the implantation process, the selected mRNAs were involved in processes important during early embryo implantation. The results showed that expression patterns of these genes were in excellent agreement with the RNA-seq findings (Table 2).

### Genomic features of lncRNAs

**Figure 2:**
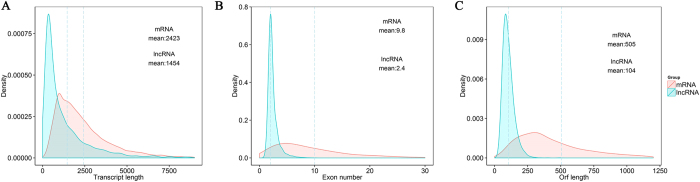
Genomic features of predicted lncRNAs. (**A**) Length distribution of 38222 coding transcripts (pink) and 2805 new predicted lncRNAs (blue). (**B**) Exon number distribution of coding transcripts and lncRNAs. (**C**) Orf length distribution of coding transcripts and lncRNAs. Dotted line represents the average value.

**Figure 3:**
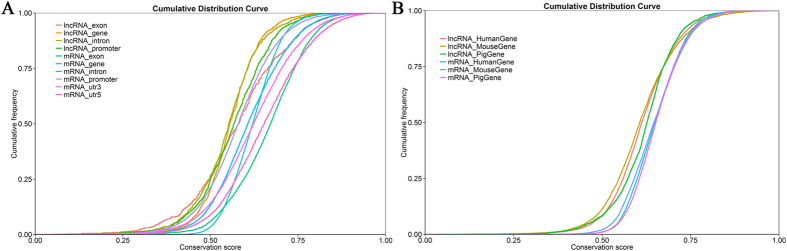
Conservation of new predicted lncRNAs. (**A**) Conservation score for 38222 coding transcripts and 2805 new predicted lncRNAs by using phasCon software. (**B**) Conservation score comparison for coding transcripts and lncRNAs in pig, human and mouse species.

Many studies have shown that lncRNAs were shorter in length, less conserved than protein coding transcripts^27^. In agreement with previous studies, our results indicated that the predicated lncRNA are shorter in length than protein coding transcripts (Fig. 2A), and their genes tend to contain fewer exon (Fig. 2B). We found that lncRNAs in endometrium were longer than in skeletal muscle (1043bp on average), the number of exon was similar^34^. Interestingly, lncRNAs in pig endometrium are shorter in length than lncRNAs in human (1 kb on average), but longer than those in mouse (550nt on average) and zebrafish (1113nt on average), and contain fewer exon number than human (2.9 exon on average), mouse (3.7 exon on average) and zebrafish (2.8 exon on average)^35^,^36^. We also found that our predicted novel lncRNAs were less conserved than protein coding transcripts by using phastCon (Fig. 3), which was similar to that of the previous reports^37^. Furthermore, the identified lncRNAs in our dataset tend to be shorter in Orf length than protein coding genes (Fig. 2C). We annotated the traits of each putative novel lncRNA, such as chromatin state, proximity to coding genes, and the relationship of location and its target genes (Supplementary Table S4).

### Differences in gene expression patterns between lncRNAs and protein coding transcripts

**Figure 4:**
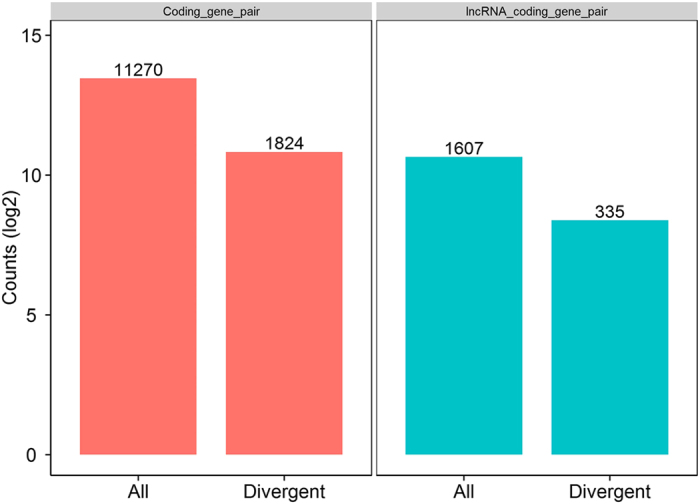
Number of gene pairs formed by lncRNAs and their neighboring coding genes. Proportion of divergent and all directions in coding gene pairs (red) and lncRNA: coding gene pairs (blue).

**Figure 5:**
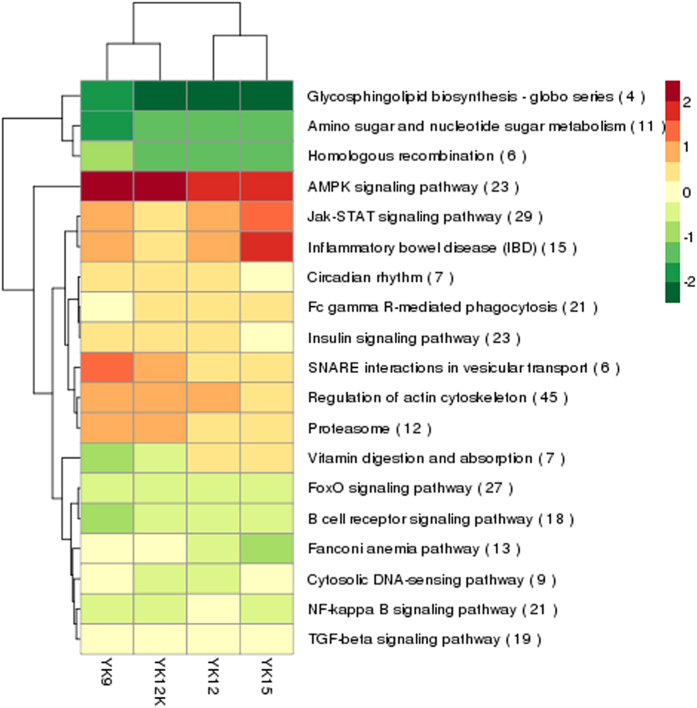
KEGG annotation for neighbor gene functions of predicated lncRNAs. Red shows higher expression and green shows lower espression. The number in the parentheses means the number of differentially expressed genes in this term.

**Figure 6:**
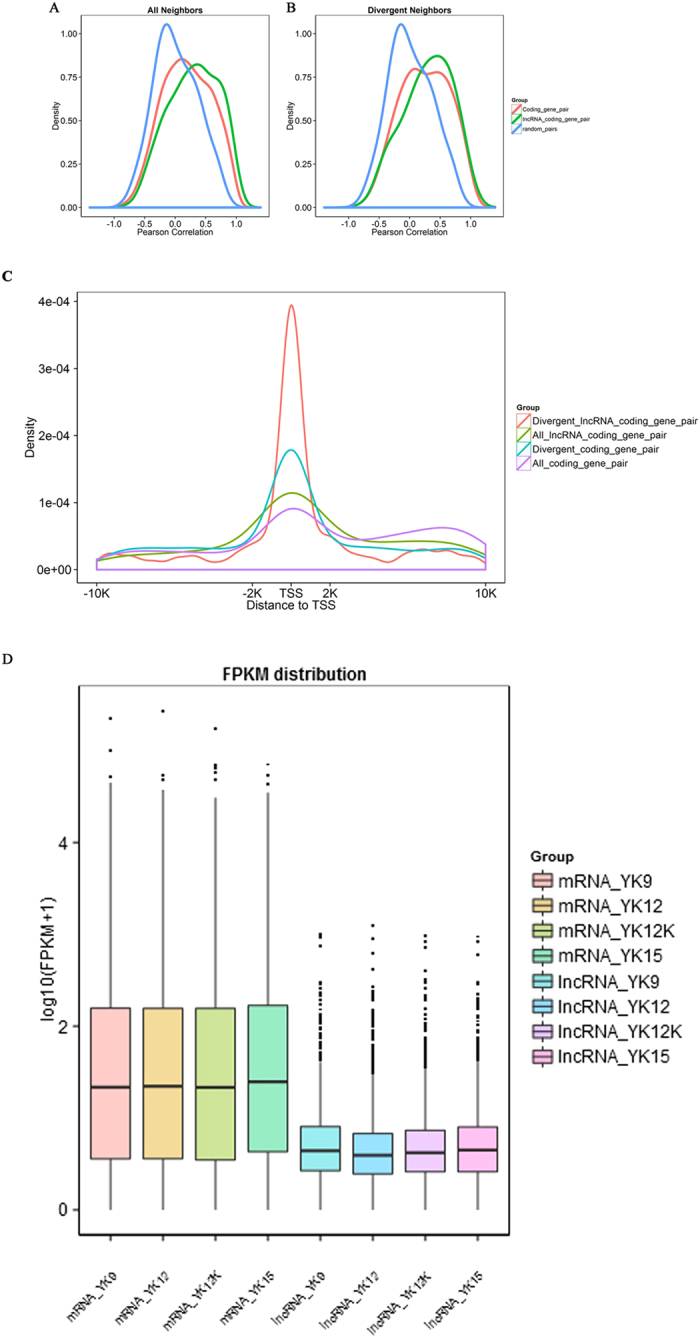
Correlation of expression patterns between pairs of neighboring genes. (**A**) Shown are distributions of Pearson correlation coefficients in expression levels between either 11270 pairs of coding gene neighbors (red), 1607 pairs of long non-coding RNAs (lncRNAs) and their neighboring coding genes (green), or random pairs of genes (blue). (**B**) Shown are distribution of Pearson correlation coefficients calculated as in A, but only for 335 pairs of divergently transcribed pairs of lncRNA ans protein-coding genes (green) or 1824 pairs of divergently transcribed protein-coding genes (red). (**C**) Distribution of distance from one TSS (transcription start sites) to another, in all directions of lncRNA: coding gene pairs (green) and coding gene pairs (purple), divergent direction of lncRNA: coding gene pairs (red) and coding gene pairs (blue). (**D**) Box plots (showing the 15th, 25th, 50th, 75th, and 95th percentiles) showing the expression feature of lncRNA and mRNA in each samples.

Recent studies suggested that lncRNAs may act in *cis* and affect the gene expression of their chromosomal neighborhood in 100k of upstream and downstream^38^. To investigate the relationship between lncRNAs and their neighboring coding genes, we analyzed gene pairs formed by lncRNAs and their neighboring genes, and identified 11270 coding gene: coding gene pairs (1824 in divergent) and 1607 lncRNA: coding gene pairs (335 in divergent) (Fig. 4). Each category of gene pairs was mainly uni-direction pairs. For these coding genes near lncRNAs, pathway of “Regulation of actin cytoskeleton”, “Jak-STAT signling pathway”, “FoxO signaling pathway” and “MAPK signaling pathway” were enriched (Fig. 5). We observed a more correlated expression pattern of lncRNAs with their neighboring gene pairs (mean correlation: 0.297) than random coding gene pairs (mean correlation: 0.019) (*P*-value < 2.2e-16, Kolomogorv-Smirnov Test) and it exhibit a relatively higher correlated than coding gene pairs (mean correlation: 0.195) (*P*-value = 4.441e-16) (Fig. 6A). Notably, there was a significantly higher correlation between divergent lncRNAs: coding gene pairs (mean correlation: 0.294) than divergent (bidirectional) coding gene pairs (mean correlation: 0.247) (*P*-value = 0.0109) as well as random coding gene pairs (mean correlation: 0.019) (*P*-value < 2.2e-16) (Fig. 6B). This analysis suggested that the correlation between lncRNAs and their neighboring coding genes were higher than random gene pairs and coding gene pairs.

**Figure 7:**
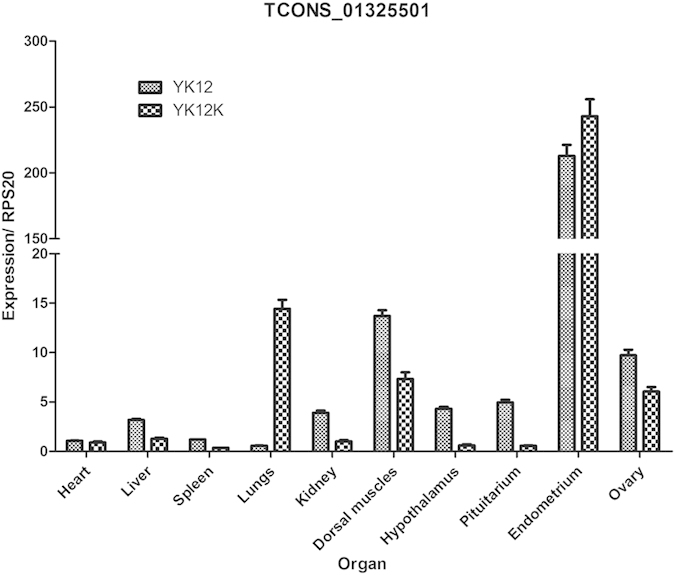
Relative expression of lncRNA. Expression profiles of lncRNA *TCONS_01325501* in ten tissues on pregnant day 12 (YK12) and non-pregnant day 12 (YK12K) pigs are expressed as the mean ± SEM.

Further analysis illustrated that many lncRNAs were located with a 4-kb region surrounding the transcription start sites (TSSs) of coding genes and the majority of these lncRNAs originate from divergent transcription of lncRNAs: coding gene pairs (Fig. 6C). This conclusion was consistent with the previous results^39^,^40^. Our results indicated that lncRNAs tend to be expressed at lower levels than mRNA (Fig. 6D). We next examined the expression of selected lncRNAs in ten tissues of pregnancy and non-pregnancy (Fig. 7). Notably, lncRNAs tended to have high expression levels in endometrium than in other tissues.

### Differential expression cluster analysis and Functional Prediction of LncRNAs in Endometrial Tissue Samples

To gain insight into the similarities of endometrium from Yorkshire of four ages at the transcriptome scale, data from all the differentially expressed genes in endometrium were used in a systematic cluster analysis. The heat map clearly suggested that YK12K and YK9 were initially clustered together because their expression profiles were similar, while YK12 and YK15 were clustered in another class (Fig. 1B). To further predict the function of lncRNAs in endometrial of pigs, we performed a Gene Ontology (GO) analysis with the selected mRNAs which neighbor lncRNAs or have high correlations with the expression of lncRNAs in 4 comparison groups. GO terms with the highest number of DGEs were related to remodeling of the endometrium, such as “binding”, “cellular process”, “immune system process” and “multicellular organismal process” (Supplementary Fig. S3). Furthermore, significantly enrichment GO terms (corrected p-Value < 0.05) were mainly including “multicellular organismal process” and “MHC class II protein complex” (Supplementary Table S5). The KEGG analysis revealed that the significantly enriched pathways during early pregnancy were “cytokine-cytokine receptor interaction”, “ribosome” and “neuroactive ligand-receptor interaction”, respectively. Interestingly, “estrogen signaling pathway” was the specific enrichment pathway in YK12 vs YK9 comparison group, and “PI3K-Akt” and “Jak-STAT” were the common pathway in the four comparison groups (Supplementary Fig. S4). To find the specific enrichment functional terms for up-regulated and down-regulated lncRNAs, separated GO and KEGG analysis were performed. All up-regulated lncRNAs in four comparisons were related to steroidogenesis, immune function, cellular component biogenesis and tissue remodeling (Supplementary Table S6). As the number of down-regulated lncRNAs was small, there were no significant GO terms enriched in the DEGs in the four comparisons, and the four most enriched KEGG pathways were “ribosome”, “allograft rejection”, “leukocyte transendothelial migration” and “glycerophospholipid metabolism” (Supplementary Table S6). In addition, the category related to “allograft rejection” term was found to be peculiar for down-regulated genes enrichment in YK15 vs YK9 group.

### Functional analysis of mRNA in endometrial tissue samples

DEGs were enrichment in biological functions of immune response and extracellular matrix in uterine endometrium for YK15 vs YK9 and YK15 vs YK12 comparisons groups (Supplementary Table S7), but there were no GO terms enrichment for other two groups. To identify the signal transduction pathways in these four comparisons, we performed analysis on the basis of the KEGG pathway database. It was observed that the common signaling pathways was “PI3K-Akt signaling pathway” in YK12 vs YK9, YK15 vs YK12 and YK15 vs YK9 comparisons groups (Supplementary Fig. S5, S6).

## Discussion

The majority of embryonic loss has occurred primarily around Day 11–12, and the maternal recognition of pregnancy signal around Day 12 of gestation^41^,^42^. At mRNA expression levels, a large number of high throughput platforms have been performed to analyze the endometrial mechanisms and pathways around Day 12 of gestation^20^,^21^. The analysis of gene expression merely on mRNA levels has become feasible. With the development of high-throughput technologies for large-scale expression feasible, RNA-seq has accelerated the discovery and characterization of lncRNAs, a new class of biologically-significant RNA transcripts^30^,^35^. However, to our knowledge, lncRNAs in endometrium tissue is little known. As for the first study of lncRNAs in endometrium of pigs, we identified approximately 2806 putative noncoding transcripts and 2,376 (301 lncRNA and 2075 mRNA) DEGs. 36 up-regulated and 38 down-regulated DGEs were obtained from YK12 compared with YK12K. However, previous study have found that 1335 with higher and 1258 with lower expression levels of DEGs from pregnant gilts compared to the non-pregnant controls. This may caused by difference between species.

The primary goal of this study was to identify non-coding RNA, mainly lncRNA. Our newly identified lncRNAs in pig endometrium shared many characteristics with those in other mammalian species. They are shorter, lower in exon number, lower in expression level and less conserved than protein coding transcripts. Furthermore, the conservation of lncRNAs in pig was modestly lower than in human and mouse. In particular, recent studies demonstrated that some lncRNAs can regulate gene expression of their chromosomal neighborhood in *cis*^43^,^44^. Non-coding RNA transcripts can also be derived from divergent transcripts in mammals^45^,^46^, and the RNAs produced by divergent transcription may serve a regulatory function for expression of the downstream genes^47^. According to the analysis of the gene pairs formed by lncRNAs and their neighboring genes, we found lncRNAs transcripted coordinately with neighboring genes and at a higher level than the neighboring coding gene pairs. The majority of lncRNAs are divergent transcription of active protein coding genes. As a contrast of this, some other researchers suggested that pairs of coding gene neighboring are slightly more correlated to each other than neighboring lincRNA: protein-coding gene pairs^35^.

In this study, lncRNAs mainly included long intergenic noncoding RNA (lincRNA), inronic lncRNA and anti-sense lncRNA. Previous studied have noted that lincRNAs can be classified as enhancer-associated (elncRNA) or promoter-associated (plncRNA)^48^,^49^. A notable feature of the lncRNAs is remarkable tissue-specific as compared with protein-coding genes^50^,^51^, both elncRNA and plncRNAs exhibit tissue specificity^32^. So we verified selected lncRNAs used for validating the RNA-seq results in difference tissues and got the similar conclusion, RNA expression profiling across pig tissues revealed that the transcript *TCONS_01325501* was highly expressed in endometrium.

Most evidence suggests that the expression of lncRNAs can regulate and have high correlations with expression of neighboring mRNAs^35^,^52^. Based on this, we searched coding genes 10k/100k upstream and downstream of lncRNA as the *cis* target genes and predicted the function of lncRNA. Consequently, we found that many lncRNAs may exert their function through predicted mRNA which can play pivotal roles in pig endometrium during pre-implantation phase. For example, the sequence of lncRNA *TCONS_01325501* matched with *FGF7*, *FGF7* has been reported to stimulate cell proliferation, differentiation, migration, and vascular angiogenesis^12^,^53^. This finding suggests that *TCONS_01325501* may affect the expression of *FGF7* and therefore be involved in the interaction between the uterus and conceptus. In this study, lncRNAs of *TCONS_01729386* was the only identified different transcripts in YK12 vs YK9, YK15 vs YK12 and YK15 vs YK9 comparisons groups, its predicted target mRNA were *FGF9* and *IL-1.* The role of *IL-1* and *FGF9* during pre-implantation phases is important for established of pregnancy. *FGF9* was significantly higher expressed in pregnant animals, in line with the fact that *FGF9* has previously been identified as a growth factor in pig endometrium^54^. *IL-1* was identified as one such paracrine factor that modulates the communication between the maternal endometrium and embryo^16^,^55^. Therefore, we speculated that *TCONS_01729386* may regulate the embryo implantation. However, these predicted functions of lncRNAs require experimental verification.

The most critical period during implantation in pigs is considered to be Day 12 of pregnancy, the time when maternal begins to recognize of pregnancy^7^. One of the most important finding of the study was that in the four comparison groups we identified 5 genes, namely *LOC100153672*, *NMB*, *LOC100622067*, *S100A8* and *JPH1*, which were specific up-regulated expression genes in YK12 sample compared to YK9, YK15 and YK12K samples (see Supplementary Table S5, Supplementary Table S6). GO functional annotation analysis showed that they were all involved in the “protein binding” and “extracellular matrix” terms, and were related to “PI3K-Akt signaling pathway”, “MAPK signaling pathway”, “Protein digestion and absorption” and “Insulin signaling pathway”. Other eight genes, namely *FGF7, C7, COL5A3, ZFYVE28, USH1C, PPP1R3D* and *EPS8L3* were up-regulated in YK12 samples as compared to YK9 and YK15 samples, which enriched of GO terms related to protein binding and transcription factor activity. *S100A8* is low molecular weight calcium binding protein and is found at high level in the extracellular milieu during inflammatory conditions^56^, while another study found that up-regulation of *S100A8* is a key component of the early endometrial response to uterine infection^57^. *PPP1R3D* is clustered into the pathway of insulin signaling pathway, which is a possible influential factor of blood vessel development. Apposition and attachment of pig conceptuses need a maternal vascular support. Estrogens are pivotal for maternal recognition of pregnancy in pigs. *FOXO1* is known to be regulated by steroid hormones, including estrogen and progesterone, which is up-regulated in the uterine endometrium on Day 12 compared with Day 9 of pregnancy^58^. Other important finding was “Estrogen signaling pathway” which as the specific pathway in YK12 vs YK9 group, this may due to the highest level on Day 12 of pregnancy than other periods. *PIK3R3* and *ESR1* were the DEGs in this pathway, also we found that *PIK3R3* was in the other two pathways, “Jak-STAT” and “PI3-Akt”. *IL6R*, *IL5*, and *IL10RB* were enriched in “Jak-STAT” pathway, *EGF* and *IL6R* were enriched in “PI3-Akt” pathway.

All up-regulated lncRNAs in four comparisons were related with steroidogenesis in this study. Early pregnancy is accompanied by many immune reactions simultaneously occurring in the uterus^16^. GO annotation analysis indicated that the activation of immune system was the strongest at Day 15 of pregnancy. Previous study have shown that sows with lower immune system activation are prone to implantation^59^. Mitosis-related genes, such as *FGF7*, *FGF9*, *IGFBP2*, *MET* and *MKI67*, are up-regulated in endometrium on Day 12 of pregnancy. Previous study suggested that *MUC4* was significantly up-regulated in the pregnant endometrium, which indicated that *MUC4* played a vital role in protecting the porcine surface epithelium against invasion of the conceptuses. In this study, the expression of *MUC4* was significantly higher on Day 12 than on Day 9 of pregnancy.

In conclusion, we first generated the expression profile of lncRNA in pig endometrium based on a transcriptome RNA-seq approach. We have identified lncRNA and mRNA expression profile for pig endometrium on Days of 9, 12, 15 of pregnancy and Day 12 of non-pregnancy in Yorkshire pigs. Importantly, we analyzed the genomic feature and expression profiles of all identified lncRNAs. Bioinformatic analysis suggests that some lncRNAs are involved in important biological processes associated with embryo implantation such as binding, cell adhesion and growth factor activity and may play an important role in regulating the gene expression of pre-implantation. As the role of lncRNAs in pigs have not yet been fully identified and understood, this study should provide valuable resource for further studies. This study also provided a resource for lncRNA studies in other noninvasive animal implantation.

## Materials and Methods

### Ethics Statement

All studies involving animals were conducted according to the regulation (No. 5 proclaim of the Standing Committee of Hubei People’s Congress) approved by the Standing Committee of Hubei People’s Congress, P. R. China. Sample collection was approved by the ethics committee of Huazhong Agricultural University. Animals were humanely sacrificed as necessary to ameliorate suffering.

### Sample collection

Twenty Yorkshire gilts with similar age and genetic background from one commercial herd were selected. Animals were randomly assigned to cyclic (n = 3) and pregnant (n = 9) group. Gilts of pregnant group were artificially inseminated twice after estrus. Uteri were obtained from animals slaughtered on Days 12 (n = 3) of the estrous cycle or Days 9 (n = 3), 12 (n = 3) and 15 (n = 3) of pregnancy, each uterine horn was flushed with PBS (pH 7.4), and subsequently opened longitudinally at the inner side. Samples from the endometrium of the pregnant and non-pregnant sows were taken from three locations of each uterine horn: proximal, medial and distal. Tissue samples were frozen in liquid nitrogen and stored at −80 °C until RNA was isolated.

### Total RNA isolation

Total RNA was isolated from each individual sample using TRIzol reagent (Invitrogen, USA). Purity and quantity of total RNA were measured by using Nanodrop equipment. Integrity of RNA was assessed using the RNA Nano6000 Assay Kit of the Bionalyzer 2100 system (Agilent Technologies, CA, USA)

### Library preparation for lncRNA sequencing

A total amount of 3 μg RNA per sample was used as input material for the RNA sample preparations. Firstly, ribosomal RNA was removed by Epicentre Ribo-zero™ rRNA Removal Kit (Epicentre, USA), and rRNA free residue was cleaned up by ethanol precipitation. Subsequently, sequencing libraries were generated using the rRNA-depleted RNA by NEBNext^®^ Ultra™ Directional RNA Library Prep Kit for Illumina^®^ (NEB, USA) following manufacturer’s recommendations. First strand cDNA was synthesized using random hexamer primer and M-MuLV Reverse Transcriptase. Second strand cDNA synthesis was subsequently performed using DNA Polymerase I and RNase H. In the reaction buffer, dNTPs with dTTP were replaced by dUTP. After adenylation of 3′ ends of DNA fragments, NEBNext Adaptor with hairpin loop structure were ligated to prepare for hybridization. In order to select cDNA fragments of the preferred 150~200 bp in length, the library fragments were purified with AMPure XP system (Beckman Coulter, Beverly, USA). At last, products were purified (AMPure XP system) and library quality was assessed on the Agilent Bioanalyzer 2100 system. The libraries were sequenced at the Novogene Bioinformatics Institute (Beijing, China) on an Illumina Hiseq 2500 platform and 100 bp paired-end reads were generated.

### Data analysis

Raw reads of fastq format were firstly processed through in-house perl scripts. In this step, clean data (clean reads) were obtained by removing reads that contain adapter or ploy-N and low quality reads from raw data. At the same time, Q20, Q30 and GC content of the clean data were calculated. All the downstream analyses were based on the clean data with high quality. Reads were mapped with Tophat (v2.0.9) to the porcine genome sequence assembly (Sscrofa 10.2). The mapped reads of each sample were assembled by both Scripture^27^ and Cufflinks^33^.

### Coding potential analysis and Target gene prediction

We used CNCI, CPC, PFAM and phyloCSF to distinguish mRNA from lncRNA. CNCI profiles can effectively distinguish protein-coding and non-coding sequences independent of known annotations by adjoining nucleotide triplets^60^. CPC searches the sequences with known protein sequence database to clarify the coding and non-coding transcripts mainly through assessing the extent and quality of the ORF in a transcript^61^. Each transcript can be translated in all three possible frames and Pfam Scan (v1.3) used to identify occurrence of any of the known protein family domains documented in the Pfam database^62^. PhyloCSF examines evolutionary signatures characteristic in alignments with conserved coding regions, such as the high frequencies of synonymous codon substitutions and conservative amino acid substitutions, and the low frequencies of other mis-sense and non-sense substitutions to distinguish protein-coding and non-coding transcripts^63^.

Transcripts without coding potential were our candidate set of lncRNAs. Then, we searched coding genes 10k/100k upstream and downstream of lncRNA as the *cis* target gene. Trans role of lncRNA is to identify each other by the expression level.

### Conservative analysis

The phast software (v1.3) generally used for phylogenetic analysis and thus phastCons expression. PhastCons is a conservation scoring and identifying program of conserving elements. We used phyloFit to compute phylogenetic models for conserved and non-conserved regions among species and then set the model and HMM transition parameters for phastCons to compute the conservation scores of lncRNA and coding genes^64^.

### GO and KEGG enrichment analysis

The quantification of both lncRNAs and coding genes in each sample was calculated by Cuffdiff (v2.1.1)^27^, and transcripts with a *P*-adjust < 0.05 were assigned as being differentially expressed. KEGG is a database resource for understanding high-level functions and utilities of the biological system, so we used KOBAS software to test the statistical enrichment of differential expression genes or lncRNA target genes in KEGG pathways.

### Real-time RT-PCR

RNA samples from the 12 animals used for the RNA-seq experiment were analyzed by qPCR. Total cDNA was synthesized using reverse transcriptase Kit (TaKaRa, Dalian). QPCR were performed using LightCycler 480II Real-Time PCR System and SYBR^®^ Green PCR Master Mix (TaKaRa, Dalian). Each real-time RT-PCR reaction (in 25 μL) involved 12.5 μL 2 × SYBR Green Realtime PCR Master Mix (TaKaRa, Dalian), 1 μL of each primer, 2 μL cDNA and 8.5 μL H_2_O. The cycling conditions included an initial single cycle (95 °C for 3 min), and followed by 40 cycles (95 °C for 15 s; 57 °C for 15 s; 72 °C for 20 s). All amplifications were followed by dissociation curve analysis of the amplified products. Specific primers were designed using the NCBI, specificities were confirmed with BLAST and gene expression levels were normalized with *RPS20* to attain the relative expression by using 2 ^(−ΔΔCt)^ value methods (Table 1). The statistical difference in gene expression was analyzed by SAS during different endometrium development stages of pregnancy. The correlation between the results of RNA-seq and qPCR was calculated using correlation test.

## Additional Information

**How to cite this article**: Wang, Y. *et al.* Analyses of Long Non-Coding RNA and mRNA profiling using RNA sequencing during the pre-implantation phases in pig endometrium. *Sci. Rep.* **6**, 20238; doi: 10.1038/srep20238 (2016).

## Supplementary Material

Supplementary Figures

Supplementary Table S1

Supplementary Table S2

Supplementary Table S3

Supplementary Table S4

Supplementary Table S5

Supplementary Table S6

Supplementary Table S7

## Figures and Tables

**Table 1:** Number of differentially expressed genes in each comparison.

Genes	YK12 vs YK9	YK15 vs YK12	YK15 vs YK9	YK12 vs YK12K
Up regulated	466	104	1095	38
Down regulated	418	65	1023	25
Total	884	169	2118	63

**Table 2:** Validation of RNA-seq results by using quantitative RT-PCR.

Acession No.	Genes	YK9	YK12	YK12K	YK15	correlation
TCONS_01729386	RPKM	0.08	2.62	0.11	8.03	1
	QPCR	0.96	72.7	2.08	216.58	
TCONS_01325501	RPKM	13.93	419.96	405.59	156.74	0.79
	QPCR	0.83	10.91	25.5	8.15	
FGF7	RPKM	64.16	1661.11	1941.38	606.82	0.94
	QPCR	0.8	16.28	29.52	9.48	
NMB	RPKM	0.25	18.64	7.26	31.62	0.95
	QPCR	1.3	233.4	151.9	291.2	
FGF9	RPKM	42.61	124	82.38	133.1	0.7
	QPCR	0.96	1.87	2.2	2.91	
VEGFC	RPKM	2.13	45.57	6.22	65.58	1
	QPCR	1.12	31.72	3.64	42.09	
VEGFA	RPKM	57.04	4775.03	735.11	518.18	0.98
	QPCR	1.11	84.84	27.95	11.19	
Muc1	RPKM	217.67	401.08	480.27	543.69	0.84
	QPCR	0.83	1	1.79	1.72	
ESR1	RPKM	487.74	157.18	356.81	162.18	0.93
	QPCR	1.28	0.87	0.98	0.41	
RBP4	RPKM	64.16	1661.11	1941.38	606.82	0.86
	QPCR	1.14	17.66	46.68	13.54	
