# Supplementary material for: Analyses of Long Non-Coding RNA and mRNA profiling using RNA sequencing during the pre-implantation phases in pig endometrium
Source: Sci Rep. 2016 Jan 29;6:20238. doi: 10.1038/srep20238 (PMC4731748; doi:10.1038/srep20238)
Supplement: Supplementary Figures [file srep20238-s1.pdf]

Analyses of Long Non-Coding RNA and mRNA profiling using RNA sequencing during the pre-implantation phases in pig endometrium

Yueying Wang[1], Songyi Xue[1], Xiaoran Liu[1], Huan Liu[1], Tao Hu[1], Xiaotian Qiu[2], Jinlong Zhang[1]& Minggang Lei[1]

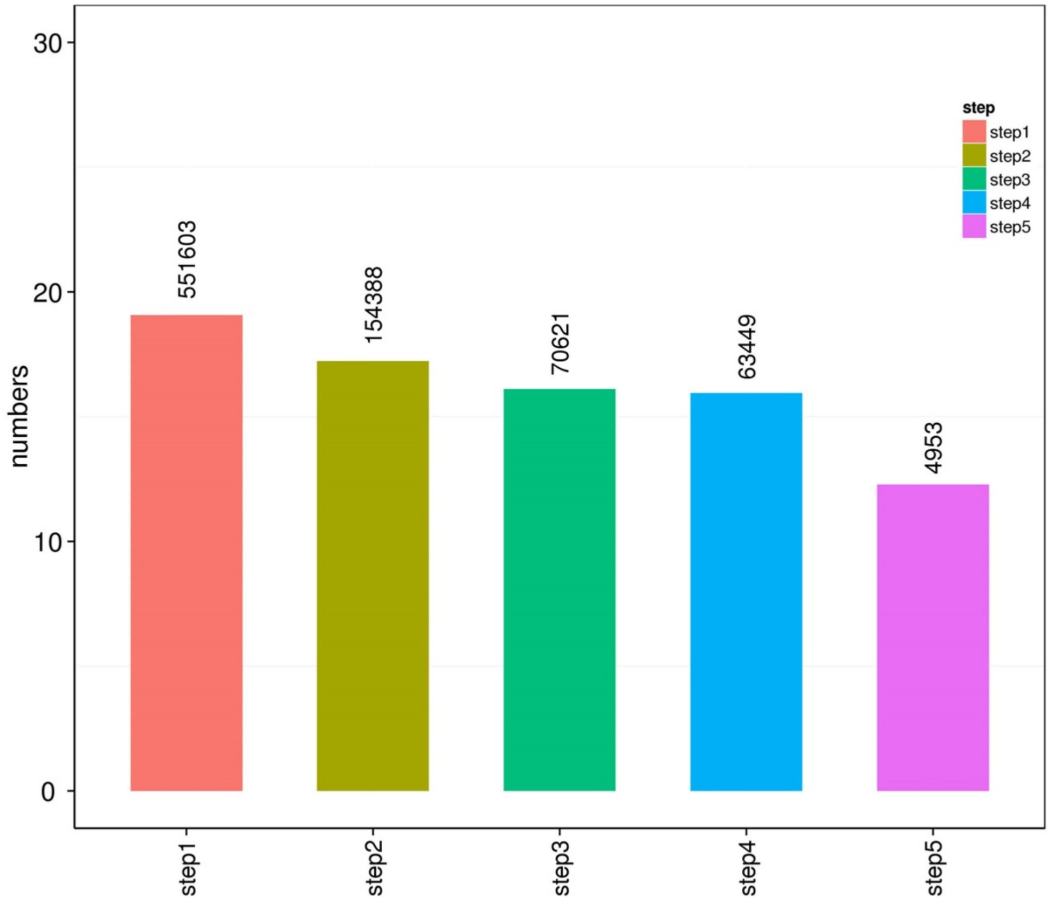

Supplementary Figure S1 4953 non-coding lncRNAs were selected by using Cufflinks and Scripture.

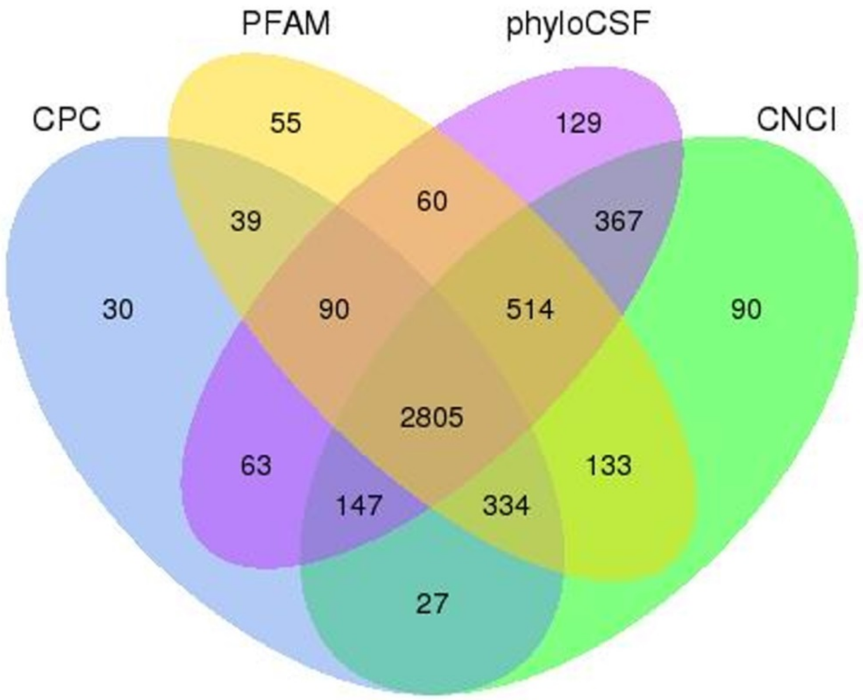

Supplementary Figure S2 Identification of non-coding lncRNAs by using CPC, PFAM, phyloCSF and CNCI. 2805 non-coding transcripts were selected by using four software evaluated protin-coding transcripts and remove putative protein-coding transcripts.

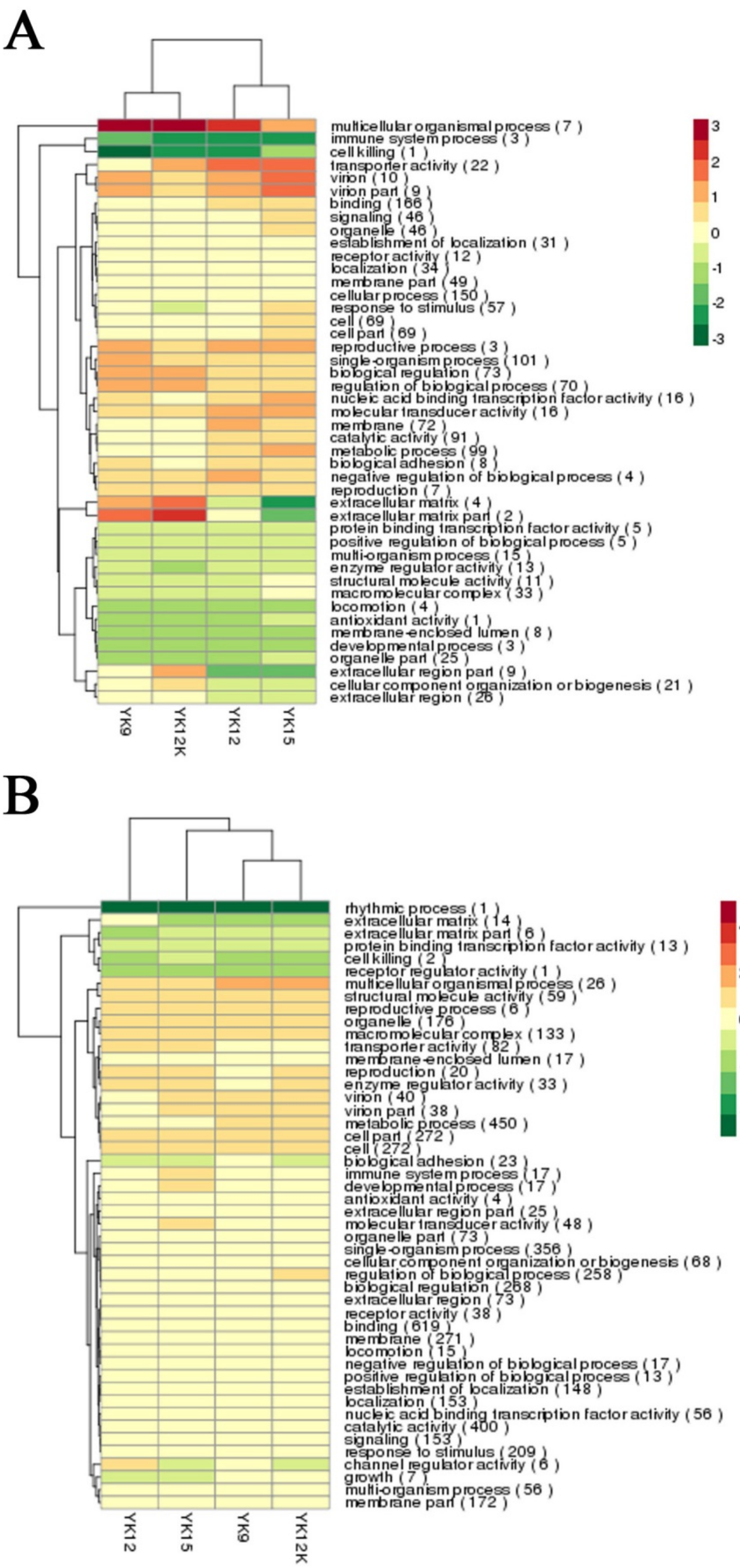

Supplementary Figure S3 GO annotation of different expression lncRNAs from four comparison groups. (A)(B) LncRNAs predicated in cis and in trans respectively, red shows higher expression and green shows lower expression.

A

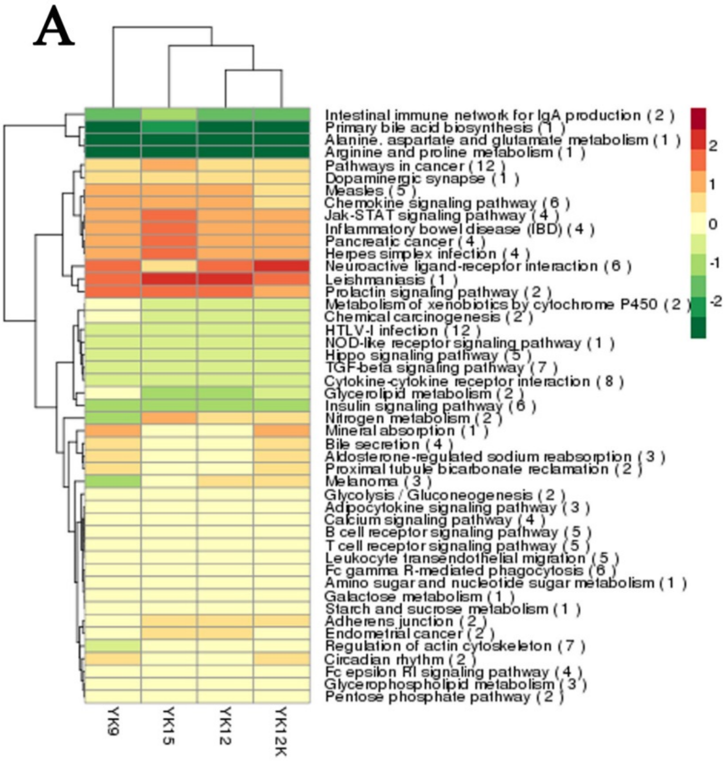

B

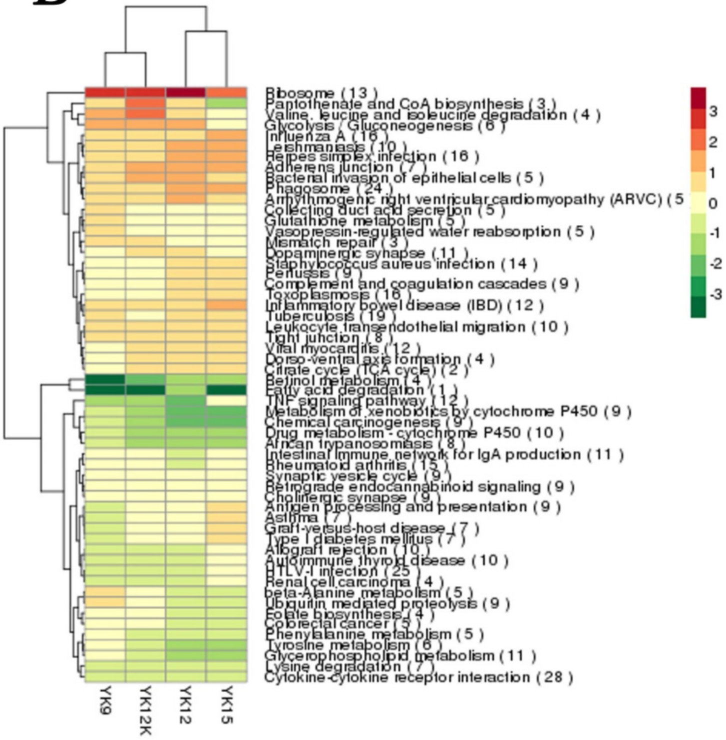

Supplementary Figure S4 KEGG annotation of different expression lncRNAs from four comparison groups. (A)(B) LncRNAs predicated in *cis* and in *trans* respectively, red shows higher expression and green shows lower expression.

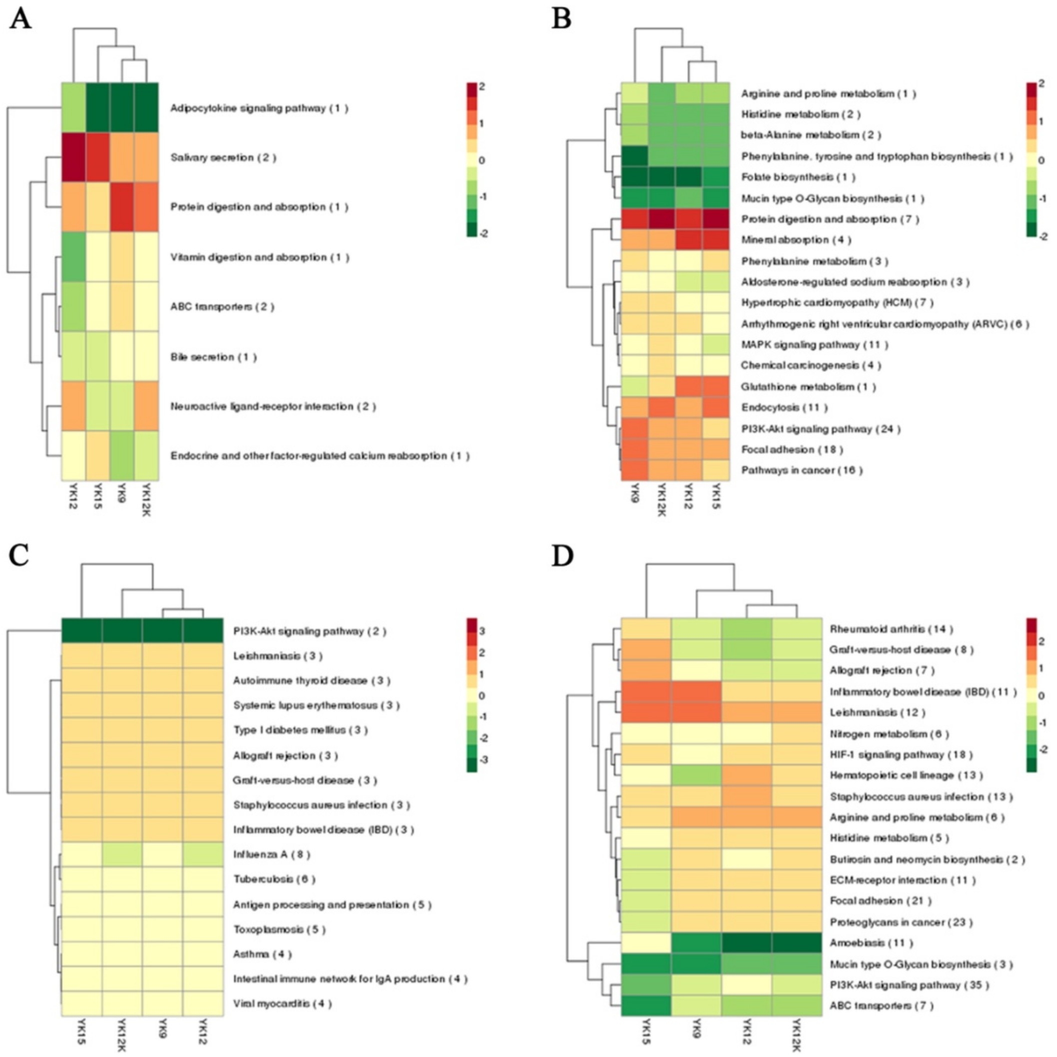

Supplementary Figure S5 KEGG annotation of up-regulated mRNAs from four comparison groups. The comparison group of YK12 vs YK12K (A), YK12 vs YK9 (B), YK15 vs YK12 (C) and YK15 vs YK9 (D), red shows higher expression and green shows lower expression. The figures in parentheses refer to the number of DGEs in this pathway. (YK: Yorkshire; YK12: day 12 of pregnancy; YK12K: day 12 of non-pregnancy; YK9: day 9 of pregnancy; YK15: day 15 of pregnancy; DGEs: differently expressed genes).

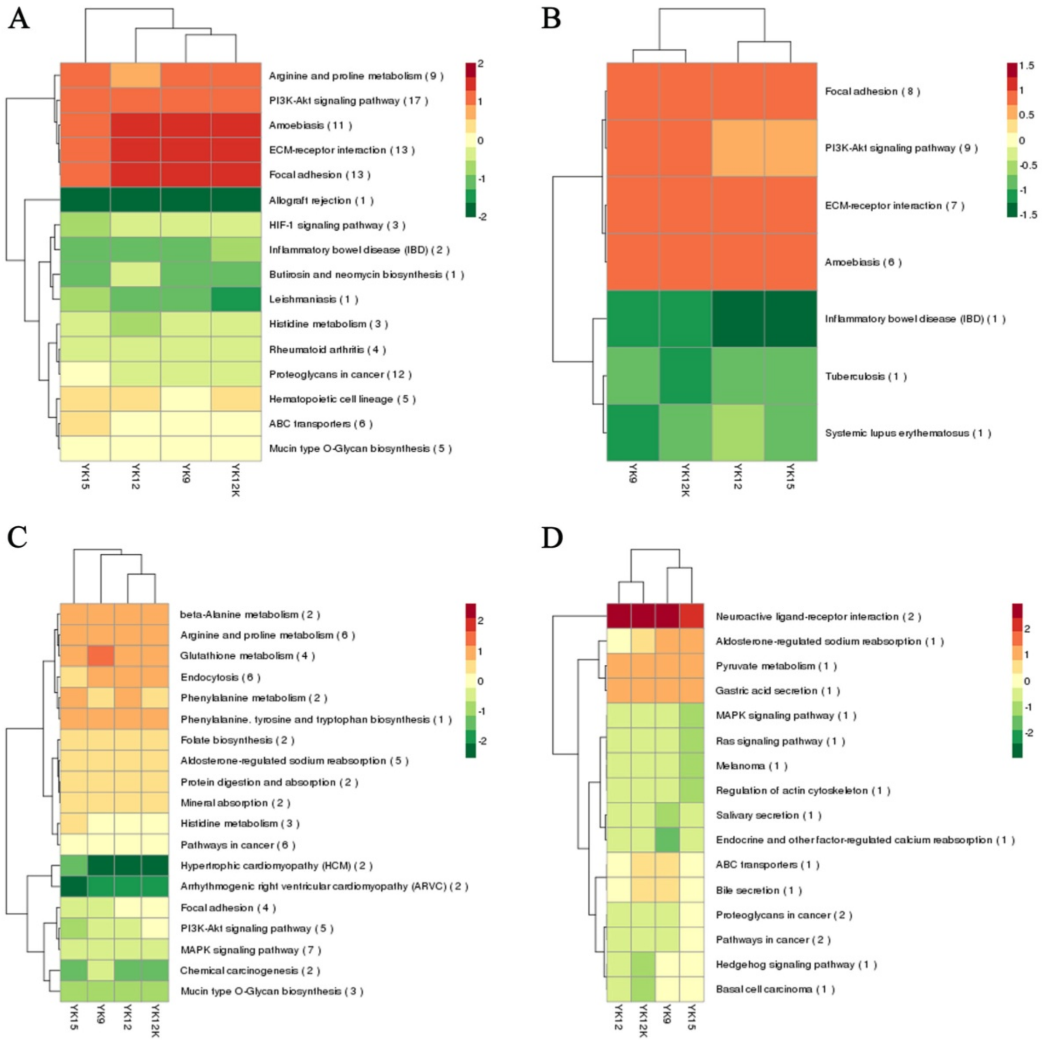

Supplementary Figure S6 EGG annotation of down-regulated mRNAs from four comparison groups. The comparison group of YK12 vs YK12K (A), YK12 vs YK9 (B), YK15 vs YK12 (C) and YK15 vs YK9 (D), red shows higher expression and green shows lower expression. The figures in parentheses refer to the number of DGEs in this pathway. (YK: Yorkshire; YK12: day 12 of pregnancy; YK12K: day 12 of non-pregnancy; YK9: day 9 of pregnancy; YK15: day 15 of pregnancy; DGEs: differently expressed genes).
